# 308. Development and Validation of a LASSO-based Score to Predict 1-year Mortality After Discharge in Patients with Severe Pneumonia Admitted to the Intensive Care Unit

**DOI:** 10.1093/ofid/ofae631.098

**Published:** 2025-01-29

**Authors:** Esteban Garcia-Gallo, Sara Duque-Vallejo, Natalia Sanabria-Herrera, André Emilio Viñán Garcés, Oriana Narváez - Ramírez, Paula Camila Ramirez Valbuena, Luis Felipe F Reyes

**Affiliations:** Pandemic Sciences Institute, University of Oxford, Oxford, UK, Oxford, England, United Kingdom; ISARIC Pandemic Sciences Institute, University of Oxford, Bogota, Distrito Capital de Bogota, Colombia; Clínica Universidad de La Sabana, Chía, Colombia, Bogota, Distrito Capital de Bogota, Colombia; Unisabana Center for Translational Science, School of Medicine, Universidad de La Sabana, Chía, Colombia, Chía, Cundinamarca, Colombia; Universidad de la Sabana, Bogota, Distrito Capital de Bogota, Colombia; Universidad de la Sabana, Chía, Colombia, Bogota, Distrito Capital de Bogota, Colombia; Universidad de La Sabana, Chía, Cundinamarca, Colombia

## Abstract

**Background:**

Severe community acquired pneumonia(sCAP) is the leading cause of ICU admission around the globe and is responsible for more than 3.5 million deaths yearly. Up to 40% require ICU admission and present a mortality rate up to 50%. Many risk factors for long-term mortality have been previously identified; however, current CAP severity scores do not discriminate patients at higher risk to die 1-year after the sCAP episode.

**Figure 1.**
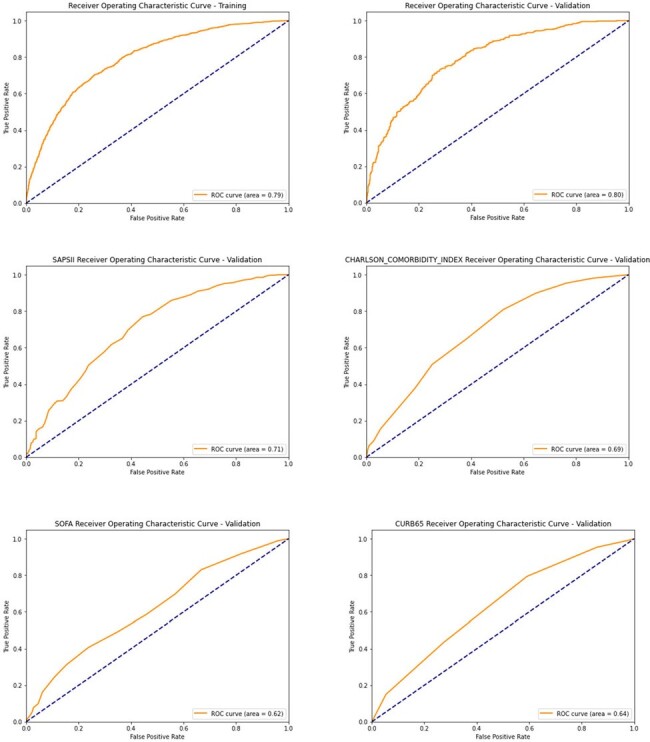
Comparative Analysis of Severity Score AUCs: Evaluating Discriminatory Power Across Metrics. A) Training B) Validation C) SAPS II D) Charlson Comorbidity Index E) SOFA F) CURB-65

**Methods:**

In this retrospective cohort study, data of 4748 adult patients with sCAP were obtained from the Medical Information Mart for Intensive Care IV(MIMIC-IV) 2001-2012 database. Univariate logistic regression analysis was used to identify those variables that tend to have a significant impact on 1-year mortality. Then a multivariate analysis was carried out to identify variables associated with one-year mortality in SCAP patients. Finally, a predictive model was developed and compared with the widely used severity of disease classification system SAPS-II. 10-fold cross-validation method was used to assess the performance metrics. The data were divided into training and validation sets in a 4:1 ratio. The least absolute shrinkage and selection operator (LASSO) regression method was used to screen the variables and to test the accuracy of the risk prediction model.

**Results:**

17 key factors were used as independent risk factors for 1-year mortality to construct the prediction model, including age, heart rate, temperature, Glasgow coma scale, malignant cancer, severe liver disease, ARDS, BUN, INR, Prothrombin Time, bilirubin, hemoglobin, urine output, supplementary oxygen, vasopressors, piperacillin tazobactam and ceftriaxone prescription. The AUC values of the prediction training and validating model were superior to the SAPS II, Charlson Comorbiditiy index, the SOFA, and CURB-65 scores [0.79, 0.80, 0.71, 0.69, 0.62, and 0.64, respectively] (Figure 1).

**Conclusion:**

Our prediction model could effectively predict 1-year mortality in patients with sCAP; additionally, it exhibits commendable classification accuracy within the ICU patient population underscoring its potential utility as a valuable prognostic tool for clinical decision-making in critically ill individuals.

**Disclosures:**

**All Authors**: No reported disclosures

